# REASONS TO REDEFINE MORAL DISTRESS: A FEMINIST EMPIRICAL BIOETHICS ANALYSIS

**DOI:** 10.1111/bioe.12783

**Published:** 2020-07-12

**Authors:** Georgina Morley, Caroline Bradbury‐Jones, Jonathan Ives

**Affiliations:** ^1^ Center for Bioethics Cleveland Clinic USA; ^2^ School of Nursing University of Birmingham Birmingham United Kingdom of Great Britain and Northern Ireland; ^3^ Centre for Ethics in Medicine University of Bristol United Kingdom of Great Britain and Northern Ireland

**Keywords:** clinical ethics, clinical practice, empirical bioethics, feminist ethics, moral distress, nursing ethics

## Abstract

There has been increasing debate in recent years about the conceptualization of moral distress. Broadly speaking, two groups of scholars have emerged: those who agree with Jameton’s ‘narrow definition’ that focuses on constraint and those who argue that Jameton’s definition is insufficient and needs to be broadened. Using feminist empirical bioethics, we interviewed critical care nurses in the United Kingdom about their experiences and conceptualizations of moral distress. We provide our broader definition of moral distress and examples of data that both challenge and support our conceptualization. We pre‐empt and overcome three key challenges that could be levelled at our account and argue that there are good reasons to adopt our broader definition of moral distress when exploring prevalence of, and management strategies for, moral distress.

## 1 CONCEPTUAL DEVELOPMENT OF MORAL DISTRESS

When Jameton introduced moral distress (MD) to the nursing literature, he suggested that moral judgement and constraint were both necessary and sufficient conditions for MD, stating that MD only occurs ‘when one knows the right thing to do but institutional constraints make it nearly impossible to pursue the right course of action’.^1^ Since Jameton, quantitative tools and scales have been developed to test and measure MD amongst nurses and other healthcare professionals (HCPs). Qualitative studies have provided experiential accounts of MD and more recently within the theoretical literature, exploration of the concept itself has occurred.

As debates about MD have continued, two groups of scholars have emerged: those who agree with Jameton’s ‘narrow definition’ (as Fourie^2^ has coined it), and those who argue Jameton’s definition is insufficient and needs to be broadened.^3^ We have outlined many of the major conceptual problems in a review of the literature.^4^ Despite the continued debate about what it means to experience MD, there remains a lack of empirical research and conceptual literature dedicated to exploring the concept itself. We utilized an empirical bioethics methodology to explore UK nurses’ understanding of MD through a combination of empirical investigation, theoretical reflection and ethical argument. We have reported on the empirical data that underpins this analysis more fully in a previous paper.^5^ In this paper, we draw upon these data in a necessarily focused way to inform our ethical analysis and draw conclusions about how MD should be understood.

We chose feminist empirical bioethics as our methodology because feminist scholarship aims to uncover the social, political, physical and power differentials that are pivotal within everyday life and impact not only on which ethical concerns are deemed to be important but also how moral deliberation plays out.^6^ Feminist ethics developed as a movement against traditional moral philosophy, in which the moral agent is viewed as an autonomous actor, rationally deliberating from universal, abstract principles about the ‘right’ thing to do and ‘unburdened by the non‐ideal constraints of luck (moral and otherwise), circumstance and capability’.^7^ Issues related to power, justice and constraint have emerged in previous literature as central to the experience of MD^8^ and we wanted to ensure our methodology would allow us to be sensitive to these issues if they also emerged in our analysis. This approach also had practical benefits which will be highlighted in our analysis. First however, we will outline our overall methodological approach, before briefly summarizing already published work that provides the foundation of this final stage of theorizing.

## 2 METHODOLOGY

The theoretical underpinning of the project was feminist empirical bioethics because feminist ethical theory was combined with empirical data to inform conceptual development and normative recommendations. Feminist interpretive phenomenology was the methodology used to collect and analyse the empirical data which is presented and described in a different paper.^9^ Ives’^10^ method of ‘reflexive balancing’ (RBL) was used to inform the ethical analysis and enables us to clearly articulate the relationship between the empirical, theoretical, and normative in bioethics research. RBL was selected partly because it can accommodate commitment to the feminist ideals that underpinned this project from its inception, and partly because its theoretical foundations of sceptical pragmatism, modest moral foundationalism, coherence and compromise resonate strongly with the critique of current thinking on MD, in particular the need to agree upon a concept that is relevant to clinical nurses’ everyday experiences, coherent and rigorous.

RBL employs a quasi‐moral foundationalism which accommodates both the benefits of a coherentist framework of moral justification—it remains broadly egalitarian as sets of beliefs can be introduced/ rejected based on coherence—and the benefits of foundationalism, enabling a foundation from which to build our coherent belief set. Beliefs can therefore be treated as epistemically privileged and posited as true for the purposes of moral enquiry. Importantly these beliefs are only treated *as though* they are epistemically privileged, they are not actually epistemically privileged and so they can still be altered, moved or replaced. In Ives’^11^ original method, these epistemically privileged beliefs are derived from empirical data and are labelled ‘boundary principles’. Ives^12^ suggests that by deriving the boundary principles from the empirical data, this guards against researcher bias. However, we deviate slightly from this, also treating our commitment to ‘core feminism’ as a boundary principle: the commitment to seek and ‘eradicate traces of sexism and other oppressions wherever they may be found’.^13^ ‘Core feminism’ unites feminist theorists and commits them to the normative mandate of eradicating oppression.^14^ We justify this deviation on the basis that this principle is widely accepted and uncontroversial. It would be difficult to argue that it is ethically justifiable to continue the oppression of women and marginalized individuals. Further, although using this core feminist commitment as a boundary principle may introduce theoretical bias, we suggest it is a positive bias that aims for greater justice and equality, and is therefore coherent with feminist ideals.

**TABLE 1:** Steps required for reflexive balancing and steps taken in this project

Steps required for RBL*	Steps taken in this project
*Identification of a moral problem:* the problem could be rooted in practical experience, engagement with empirical literature or from theoretical considerations.	The issue of MD was first identified through GM’s experiences in practice and key questions regarding the concept raised through engagement with the empirical and theoretical literature.
*Disciplinary naïve inquiry into the problem:* this can be achieved either by data gathering, engaging with social science literature, philosophical theoretical literature, legal cases, politics and policy, and must be undertaken reflexively. Aims are twofold: To uncover and explore from multiple perspectives, all the values that operate on the problem and try to find some basic value propositions which act as quasi‐foundational boundary principles. To fully understand both micro and macro context of the problem, the way it is broadly conceived by the stakeholders, with the aim of uncovering recalcitrant experience.	Inquiry begins by systematically searching and reviewing the social science and theoretical/conceptual literature. Data gathered from stakeholders regarding their ethical experiences using feminist interpretive phenomenology. Reflexivity maintained throughout using a reflexive research diary. Systematic literature review conducted, and a hypotheses definition of MD generated to be used as a boundary principle. Stakeholders asked to describe ethical challenges and experiences of moral distress (micro), and how systems could support them (macro). Data analysed using Van Manen’s six steps and quasi‐foundational boundary principles determined (i.e. beliefs about what MD is and what causes it). Data analysed individually and collectively to uncover both shared and recalcitrant experiences.
*Reflexive balancing:* identification of boundary principles (from 2a), followed by systematically challenging those principles by actively searching for disconfirming data. If disconfirming data is found, the new boundary principle must be coherent with the others to be justified.	Hypothesis definition of MD derived from systematic review used as a starting point (boundary principle 1), and developed to make coherent with empirically identified beliefs about MD (boundary principles 2). This hypothesis account of MD was then exposed to systemic challenge from our commitment to ‘core feminism’ and other disconfirming/recalcitrant data, data from previous studies and theoretical literature. The data and theory that survives systematic challenges is used to form a coherent account of MD in UK nursing and how we ought to respond to it.

* Steps required for RBL is taken from Ives, op. cit. note 10.‘Reflexive balancing’.

Following our review of the literature (step 2), we first postulated a working definition that could be posited as a null hypothesis and used as a starting point for our ethical analysis (step 3).^15^ This definition was constructed as follows:

MD is the combination of:

- the experience of a moral event
- the experience of ‘psychological distress’

and

- direct causal relation between (i) and (ii).

This provided us with a starting point that was justifiable in light of what has already been written and argued about MD.^16^ We then explored how nurses’ lived experiences of MD might support, challenge, or help us further specify this working hypothesis. The details of this phase are published in,^17^ where details of the empirical methodology can be found, including the data analysis process and supplementary information including additional empirical data. Table 2 provides the demographic information of participants. Following this empirical work, we added the following clarifications to the working hypothesis for a more complete definition:

MD is the combination of:

- the experience of a moral event

*‘Moral event’ could be any/combination of the following: moral tension, moral conflict, moral dilemma, moral uncertainty or moral constraint*.

- the experience of ‘psychological distress’

*The term ‘psychological distress’ is an umbrella term that captures a variety of different negative emotions that may be expressed differently by each individual but the predominant emotions amongst these participants were anger, frustration, guilt, regret, sadness/upset, powerlessness, symptoms associated with stress and feeling torn*.

and

- direct causal relation between (i) and (ii).

The data supported the idea that MD is conceptualized in UK nursing practice in broad terms, giving *prima facie* reason to consider a broader definition of MD as most appropriate. At this point in the project, we have a working account of MD that appears to be conceptually coherent and consistent with the lived experiences of UK nurses as we interpreted it.

**TABLE 2:** Demographic information of participants*

**Age range**	**Number of participants**
25–34 years old	17
35–44	2
45–54	2
**Gender**	
Female	18
Male	3
**Hours of employment**	
Full‐time	18
Part‐time	3
**Primary clinical area**	
General/Trauma ITU	15
Specialist ITU	6
**Banding level**	
Junior (band 5 and 6)	12
Senior (band 7 and above)	9

Years in current role		Years registered as a nurse	
<1	3	< 1	0
1–3	10	1–3	3
3–5	6	3–5	3
5–10	1	5–10	12
10–20	1	10–20	1
20 +	0	20 +	2

**Highest qualification**	
Bachelor’s degree in nursing	12
Diploma adult nursing/nursing	1
Postgraduate diploma adult nursing	5
Other	3 1

* Also published in Morley et al. op. cit. note 5.

However, this account is still susceptible to conceptual challenges and these challenges need exploring before we can expect others to accept our account of MD. What follows is a discussion of three key pre‐emptive challenges to our account. This discussion is taken from the final stage of the RBL process, where we expose our working account to systematic challenge, and either revise it in light of that challenge, or provide a reasoned account of why it does not work.

## 3 THREE CHALLENGES

### 3.1 Challenge 1: Conceptual concerns

In this section, we will present four potential criticisms of our account based upon concerns that our definition of MD is too broad. We will present ethical justifications and a defence of our definition.

#### 3.1.1 The ‘term of art’ objection

The first proposed objection against our account is that MD is a term of art coined to capture the specific phenomenon of constrained moral judgement observed by Jameton^18^ and that we ought therefore to preserve it,^19^ the objection being that a commonsense appeal to the words ‘moral’ and ‘distress’ is not enough to resolve the issue of what MD means. However, this argument does not provide a strong justification against reconceptualizing MD if doing so would increase its utility and relevance. Our response is that we should examine the key features of MD and if we find good enough reason, we ought to reconceptualize it.

#### 3.1.2 The ‘constraint is the only cause’ objection

The second, and indeed stronger argument, is that constraint simply is the only morally relevant cause of MD and therefore we have no need to broaden Jameton’s original definition. There is much empirical evidence to suggest that moral constraint both causes and characterizes MD. The Moral Distress Scale (MDS) and Moral Distress Scale‐Revised (MDS‐R) have been used in multiple countries and contexts to capture the constraints that cause healthcare professionals to feel distressed.^20^ This is captured in the following quotation from one study participant, Rachel:

GM: I want to try and figure out, what do you think is the commonality between all of these experiences that you've had that have caused you to find them morally distressing? What do you think is at the core of it?

Rachel: I guess it's the unfairness, isn't it, in every aspect really. I think when it comes to end of life I don't think that's necessarily unfair, I just don't want people to suffer and I want to make them comfortable […] but I think the most morally distressing things are when you disagree and you just think that's wrong, you're not doing what's best for the patient.

Rachel suggests it is the unfairness associated with moral constraint that makes an experience distinctly ‘MD’. Participants spoke about feelings of injustice, anger and frustration that arose because they felt morally constrained, and they characterized these experiences as MD. However, participants also discussed other moral events that caused similar negative emotional responses. For example, another participant, Elizabeth, suggested that MD is characterized by both uncertainty and constraint.

GM: So, if I said to you, what is moral distress?

Elizabeth: I think for me it comes out of a feeling of emotional or physical distress when you either don’t feel like the right decision, well you feel like you followed a course of action or been complicit in a course of action which wasn’t the correct one or where you are unsure as to whether it was the correct one, I think it can be either or, where yeah, that’s what I would, where you’re either faced with the decision or you‘ve already done it and you are thinking that wasn’t right or I don’t know whether that was right or I think it was right but I can’t be 100% sure. It happens all the time because you can never be 100%, yeah that’s what I’d say it was.

We will examine whether we can legitimately claim that uncertainty also causes MD by comparing Elizabeth’s experience to a case study example provided by Campbell et al.^21^ of a junior surgeon who, they argued, was also experiencing MD because of uncertainty. We use this example because the arguments that Wocial^22^ and others level at this account could also potentially be levelled at our interpretation of Elizabeth’s account.

Campbell et al.^23^ provide the example of a junior surgeon who, they suggest, was uncertain about what to do when given a disproportionate caseload of complex and potentially vulnerable patients. According to Campbell et al. the junior surgeon was worried that as a new surgeon he could harm these patients and he was morally uncertain about what to do. Should he continue performing surgeries for these patients or should he raise his concerns with his seniors? Wocial^24^ argues that in this example, MD is not caused by uncertainty but by an internal constraint and therefore falls within Jameton’s original definition and does not motivate a broader understanding of MD. Wocial suggests that Campbell et al.’s example is one of confidence rather than conscience and questions whether the junior surgeon’s reluctance to do the right thing is a political rather than moral choice. Both Wocial and Epstein et al.^25^ suggest that the surgeon is not really *morally* uncertain, he just lacks the confidence to question his seniors and fears the potential repercussions of doing so. This could also be suggested of Elizabeth: she is not morally uncertain, like the junior surgeon, she just lacks confidence in her decisions, is fearful of a mistake and therefore morally distressed because she is internally constrained.

However, just because another moral agent can determine the correct course of moral action, does not mean the junior surgeon or Elizabeth could. Morreim might argue that both Elizabeth and the junior surgeon are just distressed because they are conflicted about the level of self‐sacrifice they are willing to undertake and that feeling ‘morally commandeered’ because of a constraint is different to ‘moral puzzlement’ and feelings of regret.^26^ Indeed, different moral events may cause different emotional responses; however, if these emotional responses fall under the umbrella term ‘distress’, they would fulfil our broader definition. Furthermore, if they are experiencing distress because of the conflict between the ‘right’ thing to do and self‐preservation, this again falls within our proposed broader definition and is an example of a moral conflict (moral event) between one’s personal and professional values causing psychological distress.

Wocial states:

[E]ven if the surgeon does not know exactly what is the correct course of action, he recognizes a sense of responsibility, feels powerless, is concerned for patient well‐being, and believes there is personal risk regardless of the path chosen and to do nothing simply to protect himself would compromise his integrity… His struggle represents an internal constraint and could easily fall into the current understanding of moral distress. No new definition is needed for this case.^27^

Here, Wocial is conceding that MD occurred whilst the surgeon feels morally uncertain but, she argues, because his experience already fulfils Jameton’s constraint criteria, there is no need for a new definition. However, in her theoretical paper, Fourie warns: ‘if we limit distress to cases of constraint we may be dismissing the real‐life experiences of many nurses’.^28^ We do not contest that constraint is an important cause of MD and indeed this is supported by empirical data, but we agree with Fourie that if there is sufficient evidence to suggest there are other causes of MD then we should not dismiss these experiences.

However, is there good enough justification to broaden the definition of MD to include non‐constraint experiences? Considering the boundary principles upon which our account must cohere and the commitment to attributing epistemic value to these accounts, we argue that disregarding these experiences or suggesting they are mistaken would be an act of testimonial injustice and would contribute to the oppression of these individuals. By denying that these experiences fall within the lexicon of MD, we are preventing them from making sense of their own moral experiences and associated emotions: a hermeneutic injustice (HI). Scully argues that an individual suffering HI will ultimately struggle to justify their choices and goals, make moral judgements and articulate their experiences as just or unjust.^29^ ‘In other words, through its [HI’s] effects on important features of moral agency and identity, an impoverished *epistemic* capacity is also partway to producing impoverished *moral* capacity’.^30^ To disregard these experiences as MD is, therefore, not only an epistemic wrong but a moral wrong, as we deprive these individuals of the tools to make sense of their own moral experiences. It may be argued that we can find ways to respect these reports without necessarily incorporating them into the definition of MD but the question remains, if they are not experiences of MD, then what are they?

One response could be that they are just ethically challenging experiences and moral problems that cause individuals to feel troubled and upset, and do not need to be regarded as ‘MD’. However, this response also devalues these experiences, the implication being that some experiences are not distressing enough, or are not distressing in the right way, to constitute MD. The concept of MD now has power—especially in North America—and when individuals report feeling morally distressed this implies that action should be taken to ameliorate their distress. Indeed, the power of the term can be seen in the recent responses to MD that have been developed, such as Hamric and Epstein’s system‐wide MD consultation service.^31^ If these experiences are not regarded as MD, then such services may never be established in other contexts, because (if restricted to constraint) the level of MD might be regarded as too low. Furthermore, these services would not need to address these experiences if they are not cases of MD. Latham suggests that a broader understanding of MD actually motivates a broader range of responses because it has ‘important consequences for the normative debate about what, if anything, one is obligated to do about one’s moral distress’.^32^ Indeed, there is value in the ability to label and name the experiences that many HCPs express during clinical ethics consultation.

Reflexive balancing (RBL) utilizes coherence as a standard by which to assess whether new beliefs ought to be incorporated into a body of knowledge. The most simple, justifiable and coherent answer—which values and respects moral agent experiences—is that other moral events are capable of causing MD. There is a growing body of literature that supports this conclusion and suggests we gain a fuller understanding of MD by incorporating these experiences into the definition. Fourie, for example, suggests that Jameton’s emphasis on constraint actually distorts the situation.

Fourie argues:

… a definition of moral distress, which makes constraint central to distress, seems to distort the reality of the situation. Whilst constraint may be present and its significance should not be under‐estimated […] the case does not seem to be one that is accurately portrayed as being primarily about constraint: it is not simply that other people are arbitrarily or unfairly standing in the nurse’s way but that they genuinely disagree with the nurse on a moral basis.^33^

We suggest that there are good reasons to recognize these additional causes of distress and subsequently broaden our understanding of MD to accommodate them. There remain two more criticisms of this definition on the basis of historical and conceptual concerns.

#### 3.1.3 The ‘constraint is most common’ objection

The third objection is that the most common and most distressing cause of MD is constraint and the term should be reserved only for those experiences. Indeed, moral constraint was a common cause of MD discussed by participants and, through extensive use of the MDS and MDS‐R, we have evidence to suggest that constraint causes MD in many other settings. However, we cannot say with certainty that it is the most common because other causes have not been widely accepted and thus have not been explored or measured to the same extent.

Regarding whether constraint causes the most distress, attempting to characterize MD based on severity level again disregards a great deal of moral experiences. Not only is it arbitrary to determine an MD experience based upon the severity of the distress, but it is also very difficult to measure and compare emotional experiences. Individuals react to and express their emotions in a variety of ways and it would be unfair to discount an experience of MD on the basis that it is not distressing enough to constitute MD ‘proper’.

#### 3.1.4 The ‘broadening makes it meaningless’ objection

The fourth criticism that we suggest could be levelled at this broader definition is the risk of making it ‘diagnostically and analytically meaningless’.^34^ Epstein et al.^35^ have similar worries as they stress the importance of a concept that is practical and which can help us to develop interventions to mitigate it. However, employing Fourie’s reclassification of MD into its causal constituents potentially provides a solution to this critique. Fourie suggests reclassifying narrow MD into ‘moral‐constraint distress’, MD caused by conflict as ‘moral‐conflict distress’,^36^ to which we add those identified in our empirical work: ‘moral‐uncertainty distress’, ‘moral‐dilemma distress’ and ‘moral‐tension distress’.^37^ By sub‐categorizing particular types of MD, researchers and clinicians could develop more specific measures and targeted interventions for MD. These sub‐categories are broad enough to warrant slightly different interventions but are not overly specific.

Epstein et al. note that, ‘developing interventions for various subtypes would be extraordinarily challenging—how would one develop and test an intervention for moral distress […] caused by moral uncertainty?’^38^ However, this does not provide sufficient justification for disregarding other morally relevant causes of MD, especially if ultimately these interventions are more efficient. Indeed, reporting on the system‐wide MD consultation service, Hamric and Epstein reflect upon the fact that they found MD and moral dilemmas were not always mutually exclusive.^39^ Recognizing that MD constitutes a broader range of moral events could improve such interventions.

To conclude this section, the charge that our definition is too broad is potentially the most damaging challenge. However, we have provided empirical data that suggests there are other moral causes of MD and that these other causes characterise some individuals’ MD experiences.^40^ We have provided good theoretical reasons why it is coherent to accept other causes of MD and ethical reasons why we ought to value these experiences. We have also provided practical reasons why expanding the concept may help researchers to develop more targeted responses. Importantly, we are not arguing that constraint does not cause MD, nor that broadening the definition makes moral constraint‐distress any less worthy of action. In fact, we agree with Fourie^41^ who suggests that if nurses experience disproportionate amounts of constraint distress (as seems likely considering their position in the hierarchy) then it is a matter of distributive justice that we continue trying to find ways to alleviate this distress.

### 3.2 Challenge 2: Epistemological concerns

Jameton’s and subsequent conceptions of MD have been built upon the assertion that MD only occurs when one has made a moral judgement but is constrained.^42^ In this section, we address what we anticipate to be the second substantial challenge to our broad definition—that moral judgement ought to be regarded as a necessary condition of MD. To respond to this, we first need to establish what is meant by the term ‘moral judgement’ because the terminology within the literature is currently ambiguous.

In this section, we argue that ‘moral judgement’ should be understood in a weak sense and should not be regarded as a necessary or sufficient condition of MD. The first reason for this is the variation and ambiguity regarding the way participants framed their moral judgements within their narratives. Below, we provide six excerpts from the data. In each of these excerpts, participants articulate their moral judgement in different ways:

1. ‘No matter how right I *knew* I was on a practical level, you know, seeing how it made her feel, it just, made me feel guilty’ (Beth).
2. ‘I don't *think* he should ever have been trach'ed…’ (Joyce).
3. ‘…with lots of situations there are patients that you just *think*, what are we doing?’ (Rachel).
4. ‘…you just *feel* like you’re not doing the right thing with…’ (Elizabeth).
5. ‘…you’re in *torment* and *conflict* because of the morality, the rightness or wrongness of a situation…’ (Holly).
6. ‘…it doesn’t matter about my *feelings* because it’s about the family, and it’s about the patient and what they decided and so whatever my *opinions* on the subject, they aren’t relevant…’ (Amelia),

In the first quotation, Beth suggests she knew the right thing concerning the practical issue but expressed uncertainty about the ethical issue. In the second and third quotations, Joyce and Rachel both suggested *thinking they knew* and in the fourth Elizabeth discussed *feeling* she knew. In the fifth, Holly articulates *feeling* tormented and conflicted, and seems to be uncertain; and lastly, Amelia says that her *feelings* and *opinions* do not even matter. Participants most commonly expressed their judgements in terms of empathetic feeling rather than rules or judgements, which Jaggar suggests may reflect a more feminist approach to ethics.^43^ Participants described a ‘feeling of knowing’ more akin to a moral intuition than a judgement, and they do not indicate certainty, which suggests MD can occur in a variety of epistemic states.

This variation in expression mirrors the variation in the existing definitions of MD (these can be found in.^44^ The variation and subsequent ambiguity seems to suggest that we should not take ‘moral judgement’ in its strongest sense but rather accept that MD occurs along a spectrum of epistemic strength.

The second reason we suggest moral judgement should be understood in its weakest sense is because of the complexity of clinical‐ethical decision making and prognostication. As Gallagher et al. highlight, medical decision making and prognostication are rife with uncertainty, and yet form the basis of clinical ethical decision making.^45^ If we accept that the ethical supervenes on the natural, then it seems likely that clinical uncertainty creates ethical uncertainty, and our empirical evidence suggests this results in distress.^46^ Indeed, many participants discussed the difficulties of accurate prognostication and as Elizabeth describes, she experienced distress because she felt that decision making was just guesswork—that they were gambling with other peoples’ existence:

I think the distress comes from that rock and a hard place and that’s definitely the crux of it, it’s like I don’t feel comfortable standing here and it’s been months and months and months and just I feel like I’m dragging out this family’s pain and I may be dragging out your pain to like what end… and why are we doing this? And this doesn’t seem right and this doesn’t seem fair or nice. But on the other side you’ve got what feels like sometimes a little bit of a like educated guess… those are the ones that pop up in the night you know those are the faces …and yeah it’s that rock and a hard place, it’s that gamble on someone else’s existence, well it is, you’re gambling on their existence and what state that existence will be. (Elizabeth)^47^

Other researchers have also suggested that the inherent uncertainty in prognostication and end‐of‐life decision making causes MD. Oberle and Hughes interviewed nurses and doctors in acute care areas about their perceptions of ethical problems during end‐of‐life care. They found the ‘defining feature’ of end‐of‐life decision making was uncertainty, and this was a source of MD. They stated:

…uncertainty about probable outcomes was the defining feature, leading to considerable deliberation and reflection about the ‘right thing to do’. At what point did patient suffering outweigh the probability of a positive outcome, and at what point should treatment be stopped? Even in the so‐called futile cases there remained the possibility, however slight, that a positive outcome might result from further treatment.^48^

Dzeng^49^ reflects upon interviews she conducted with physicians regarding their experience of end‐of‐life care and she describes the distress they experienced because of the use of new technologies such as extracorporeal membrane oxygenation and left ventricular assist devices that create liminal states between life and death. Dzeng describes how these physicians reported feeling unprepared to deal with these situations both clinically and ethically, and that ‘this uncertainty further contributes to moral distress’.^50^ We suggest that this acknowledgement of the uncertainty surrounding end‐of‐life care and medical prognostication should extend to a greater awareness that ethical decisions based on clinical uncertainty are likely to involve moral uncertainty, and uncertainty can itself be a cause of MD.

Some authors suggest that acknowledging MD as a sign of uncertainty can help teams to reach agreement. Reflecting on a clinical ethics case, April and April suggest that had they ‘approached our patient’s sad case through the traditional frame of moral distress, we might have concluded that we were certain the right choice was to respect the patient’s autonomy and minimize further harm’.^51^ However, interpreting their feelings of MD more broadly (as suggested by Campbell et al.^52^), they instead approached the case as one of ‘moral disagreement among sincere and well‐intentioned stakeholders’,^53^ rather than as a case of ‘the moral white knight who alone knows the right choice and struggles bravely against others’.^54^ As Johnstone and Hutchinson have argued, Jameton’s conception perpetuates nurses’ belief that their moral judgements are correct and justified suggesting that other HCPs are simply arbitrarily disagreeing and this shuts down communication and undermines the process of moral deliberation.^55^ This has the further potential of simply increasing anger and resentment between HCPs and eroding relationships, whereas acknowledging that nurses and other HCPs may experience MD when they feel torn, conflicted and uncertain could, as April and April suggest, help to bring clinicians together in shared uncertainty and reduce barriers between clinicians.^56^

Furthermore, Haidt suggests that anger is a negative moral emotion that has a narrowing effect that closes individuals off from others’ viewpoints, whereas positive moral emotions have a broadening effect that can make individuals more open to new ideas, new relationships and new possibilities.^57^ In combination with environmental and institutional changes this could help encourage dialogue between HCPs, so that as a team they can recognize the complexity of moral decision making and reach moral decisions together, thus increasing the potential for moral communities to grow. Indeed, April and April believe a broader understanding of MD helped them to foster the mutual understanding required to reach consensus.^58^

Finally, in a recent green paper, Batho and Pitton argue that ‘knowledge of the right course of action’ sets an ‘epistemic threshold’ that is too high for MD.^59^ They suggest that it is entirely plausible that the moral agent experiencing MD may feel indeterminate about the morally appropriate action, or even fail to even see the options available to her. Indeed, the moral agent may not recognize the cause of their distress, or even be able to identify the moral options available to her but, nonetheless, it still seems plausible to suggest that she feels MD.

Batho and Pitton suggest that an account of MD should avoid both this ‘epistemic threshold’ and the ‘objectivity constraint’: that the agent must be aware of all the options available to her. They suggest that many previous accounts of MD fail to recognize that MD ‘is primarily a function of how the world appears to the individual, which may be different from how the world objectively happens to be’ and that MD should not depend on ‘the world actually being as she understands it to be’.^60^ Indeed, MD is a unique phenomenon, caused and experienced differently by individuals. We ought to trust individual experiences of MD to inform the concept, as only they can provide an account of how the world appears to be to them: only a broad definition can capture these unique, individual experiences.

### 3.3 Challenge 3: Ideological concerns

Some authors have attempted to explain the concept of MD by suggesting that it occurs when one’s moral integrity is violated.^61^ Suggesting that compromised integrity is the defining feature of MD allows authors to avoid the conclusion that the moral agent knows with certainty the right thing to do, because the terminology is vague enough to capture a breadth of situations; it also allows authors to retain the spirit of Jameton’s original conception by suggesting constrained moral agency is central. However, we are reluctant to use an ill‐defined concept such as integrity to try and bring conceptual clarity because, we suggest, it only defers the problem of definition.

Batho and Pitton suggest that the notion of integrity is unclear. To gain clarity, they analysed seven accounts of MD in the existing literature which ‘endorse the claim […] that central to moral distress is the experience of loss of moral integrity’.^62^ They argue that in all seven cases studies, distress was articulated because individuals felt compromised as a person and that this feeling of compromise captures loss of integrity. However, it is also unclear what is meant by feeling compromised as a person and this account again seems only to defer the problem. Furthermore, their account can be challenged methodologically.

The authors selected accounts of MD in which all the moral agents describe situations in which they believe a moral wrong occurred. Second, the authors do not describe how they selected their accounts and, upon exploring their sources, it can be concluded that they are not representative and do not provide a broad spectrum of MD experiences. Their selected sources already seem to frame MD in a specific way, and this seems to have biased their conception of MD. One case was taken from a website which is concerned with protecting HCPs’ conscience. On the first page of the website, it states: ‘The Protection of Conscience Project supports health care workers who want to provide the best care for their patients without violating their own personal and professional integrity’.^63^ Case study 7 is taken from a paper by Hardingham^64^ who also frames MD in terms of moral integrity, and three cases were taken from a special issue of a bioethics journal edited by a prominent US MD scholar who also frames MD in terms of compromised moral integrity. Therefore, their method of case analysis is not the naïve phenomenological inquiry they suggest it is. Instead, the case studies appear to have been cherry‐picked to support a pre‐analytic understanding of the centrality of integrity to MD. Batho and Pitton^65^ have not provided a convincing argument regarding how a ‘feeling of being compromised’ clarifies MD and instead seem to have again deferred the problem.

Our secondary reason for not including integrity into our suggested definition is because the participants did not frame their experiences in this way and therefore the data did not provide any mandate to frame MD in these terms. The actual word ‘integrity’ was only referred to once amongst 21 participants and even when reviewing themes that could potentially relate to integrity such as ‘conscience’ or ‘moral compass’, these were only mentioned by three participants one or two times. Even when searching for potentially related concepts or allusions to integrity, our interpretation of the data did not indicate integrity as a theme. The theme that potentially bears the most similarities to integrity is that of personal/professional values and responsibilities. However, again, when participants discussed feeling conflicted or constrained, they framed these experiences in terms of responsibilities and values, not integrity. This suggests that amongst this group of UK nurses, experiencing MD did not necessarily entail a violation of moral integrity. Of course, it is possible that with a different, perhaps larger sample size, or a sample from a different country, moral integrity could still arise as part of the experience of MD. However, the question remains whether including moral integrity within the definition of MD really brings clarity or, as we suggest, further confuses the concept by deferring the problem of definition.

Finally, we suggest that framing compromise in this way perpetuates the belief that compromise itself is bad. Batho and Pitton^66^ indicate that feeling compromised suggests an individual is both a perpetrator and a victim, because they are unable to fulfil their deeply held beliefs. However, the ability to set one’s own values aside is often the key to achieving compromise. Reflecting on an experience shared by one participant (Max), he discussed feeling morally distressed because he was uncertain about whether he agreed with the decision to withdraw LST from a patient who was awake and had decided he wanted the IABP that was sustaining his cardiac output to be removed. Max may have felt very strongly that this was the wrong thing to do, and participating in this may have made him feel compromised as a person. However, this does not mean the decision was morally wrong for the patient. There is a very difficult balance that needs to be struck in healthcare between HCPs protecting and honouring their own values and beliefs, whilst also remaining cognizant that they have a duty of care and responsibility to patients. Framing compromise of one’s values in the way Batho and Pitton^67^ propose suggests that compromise itself is bad. An unwillingness to compromise one’s own values may make one more inflexible and reluctant to engage in the moral compromise that is often required in healthcare.

When faced with having to choose a course of action in response to a moral problem, sometimes the only way to move forward is through compromise, whether this is a compromise with others, or a setting aside of one’s own values. As Huxtable has argued:

[C]omplexity and uncertainty, both in the realm of values and in the realm of facts (as far as these can be separated), are at the centre of the case for compromise. But so too are inadequate resources and the inability to honour every competing value, coupled with a prudent desire to ensure that one's values are voiced, an ongoing relationship with one's moral opponents and the need to reach a decision on a contested issue. The circumstances are ripe for compromise when such features are present in sufficient number or scale. The achievement of a principled compromise presumes communication and negotiation between the positions available and their respective defenders.^68^

By reframing compromise in this way, it can be seen as a positive by‐product of moral decision making rather than inevitably causing distress. Again, this could also help to encourage moral communities to grow. Rather than HCPs engaging in conflict with the aim of avoiding compromise to maintain one’s integrity, individuals could instead come together with compromise as their aim.

Excluding integrity from the definition of MD is a pragmatic and coherent conclusion in line with the method of RBL. It is justified because integrity did not emerge as a finding in the empirical data, it is coherent because the inclusion of integrity provides no further conceptual clarification, and it is pragmatic because it paves the way for recognizing that compromise between HCPs, families and patients is both inevitable and potentially positive.

## 4 CONCLUSION

We have presented three possible objections to our broader definition of MD and pragmatic, justifiable and coherent responses regarding why this broader definition should be adopted. Importantly, our definition of MD is grounded in participants’ reports of MD experiences. These empirical data support theoretical arguments, also made by others, that the definition of MD should be broadened.^69^ In empirical bioethics, the aim of the researcher is not to simply accept participants accounts but to maintain a ‘critical stance’, thus enabling the formation of normative conclusions.^70^ Due to our feminist commitment to uncover and address oppressive practices, we grounded our normative arguments with these values and argued that denying these participants experiences are MD would be an act of testimonial and hermeneutic injustice. We have tried to pre‐empt the main objections to our account by using deviant cases from our data and others’ arguments in the literature. Importantly, we have argued that broadening the definition may also have practical benefits as it may help to break down barriers between healthcare professionals and help moral communities grow. For those that may argue MD (as described by Jameton) is a ‘term of art’ and broadening the concept makes it analytically and diagnostically meaningless, we suggest sub‐categorizing MD according to its cause, as suggested by Fourie. Sub‐categorizing MD into different types provides a compromise between those who think the broader conception captures their ethical experiences and those who suggest that central to MD is constraint. By striking this middle ground, we suggest that MD will not become so broad that it is meaningless, but instead we can be even more specific about the cause of distress which will ultimately enable us to develop more targeted and effective interventions to mitigate its negative effects. We suggest that a promising direction for future research would be an exploration of which types of MD seem to be most commonly experienced amongst different groups of healthcare professionals in various settings, and identifying whether targeted interventions do in fact mitigate these different types of MD.

## CONFLICT OF INTEREST

The authors declare no conflict of interest.

## Footnotes

^1^ Jameton, A. (1984). *Nursing practice: The ethical issues*. Englewood Cliffs, NJ: Prentice Hall.

^2^ Fourie, C. (2015). Moral distress and moral conflict in clinical ethics. *Bioethics,* *29*(2), 91–97.

^3^ Campbell, S. M., Ulrich, C. M., & Grady, C. (2016). A broader understanding of moral distress. *American Journal of Bioethics,* *16*(12), 2–9.

^4^ Morley, G., Ives, J., Bradbury‐Jones, C., & Irvine, F. (2019). What is ‘moral distress’? A narrative synthesis of the literature. *Nursing Ethics*, *26*(3), 646–662.

^5^ Morley, G., Bradbury‐Jones, C., & Ives, J. C. S. (2019). What is ‘moral distress’? A feminist empirical bioethics study. *Nursing Ethics*. https://doi.org/10.1177/0969733019874492

^6^ Walker, M. U. (2007). *Moral understandings: A feminist study in ethics*. USA: Oxford University Press; Jaggar, A. M. (2001). Feminist ethics. In L. C. Becker & C. B. Becker, *Encyclopaedia of Ethics* (pp. 528–539). New York: Routledge.

^7^ Gotlib, A. (2015). *Feminist ethics and narrative ethics*. Retrieved from http://www.iep.utm.edu/fem‐e‐n/

^8^ Peter, E. & Liaschenko, J. (2013). Moral distress reexamined: A feminist interpretation of nurses’ identities, relationships, and responsibilities. *Bioethical Inquiry*, *10*, 337–445; Fourie, C. (2017). Who is experiencing what kind of moral distress? Distinctions for moving from a narrow to a broad definition of moral distress. *AMA Journal of Ethics,* 19(6), 578–584.

^9^ Morley et al., op. cit. note 5.

^10^ Ives, J. (2014). A method of reflexive balancing in a pragmatic, interdisciplinary and reflexive bioethics. *Bioethics,* *28*(6), 302–312.

^11^ Ives, op. cit. note 10, p. 307.

^12^ Ibid.

^13^ Donchin, A. & Purdy, L. (1999). Introduction. *Embodying bioethics recent feminist advances*. Maryland: Rowman & Littlefield Publishers, Inc., pp. 1–13.

^14^ Ibid.

^15^ Ives, op. cit. note 10.

^16^ Morley et al., op. cit. note 4.

^17^ Morley et al., op. cit. note 5.

^18^ Jameton, op. cit. note 1.

^19^ Campbell et al., op. cit. note 3; Morreim, H. (2016). Moral distress and conflict of interest. *Americal Journal of Bioethics,* *16*(12), 27–29.

^20^ Corley, M. C., Elswick, R. K., Gorman, M. et al. (2001). Development and evaluation of a moral distress scale. *J Adv Nurs,* 33(2), 250–256; Hamric, A., Borchers, C, & Epstein, E. (2012). Development and testing of an instrument to measure moral distress in healthcare professionals. *AJOB Primary Research,* *3*(2), 1–9; Papathanassoglou, E., Karanikola, M. N., Kalafati, M. et al. (2012). Professional autonomy, collaboration with physicians, and moral distress among European intensive care nurses. *American Journal of Critical Care,* *21*(2), e41–352; Whitehead, P. B., Herbertson, R. K., Hamric, A. B. et al. (2014). Moral distress among healthcare professionals: Report of an institution‐wide survey. *Journal Nurs Scholarsh,* *47*(2), 117–125.

^21^ Campbell et al., op. cit. note 3.

^22^ Wocial, L. D. (2016). A misunderstanding of moral distress. *American Journal of Bioethics,* *16*(12), 21–23.

^23^ Campbell et al., op. cit. note 3.

^24^ Wocial, op. cit. note 22.

^25^ Ibid.; Epstein, E., Hurst, A. C., Mahanes, D. et al. (2016). Is broader better? *American Journal of Bioethics,* *16*(12), 15–17.

^26^ Morreim, op. cit. note 19.

^27^ Wocial, op. cit. note 22, p. 22.

^28^ Fourie, op. cit. note 2, p. 97.

^29^ Scully, J. L. (2018). From 'She would say that, wouldn’t she?' to 'Does she take sugar?' Epistemic injustice and disability. *International Journal of Feminist Approaches to Bioethics,* *11*(1), 106–124.

^30^ Ibid: 112.

^31^ Hamric, A. & Epstein, E. (2017). A health system‐wide moral distress consultation service: Development and evaluation. *HEC Forum,* *29*, 127–143.

^32^ Latham, S. R. (2016). Moral distress and cooperation with wrongdoing. *Americal Journal of Bioethics,* *16*(12), 31–32.

^33^ Fourie, op. cit. note 2, p. 97.

^34^ Epstein et al., op. cit. note 25.

^35^ Ibid.

^36^ Fourie, op. cit. note 2.

^37^ Morley et al., op. cit. note 5.

^38^ Epstein et al., op. cit. note 25, p. 17.

^39^ Hamric & Epstein, op. cit. note 31.

^40^ Morley et al., op. cit. note 5.

^41^ Fourie, C. (2016). The ethical significance of moral distress: Inequality and nurses' constraint‐distress. *American Journal of Bioethics,* *16*(12), 23–25.

^42^ Jameton, op. cit. note 1.; Jameton, A. (1993). Dilemmas of moral distress: Moral responsibility and nursing practice. *AWHONNS Clincial Issues in Perinatal and Womens Health Nursing,* *4*(4), 542–551.

^43^ Jaggar, op.cit., note 6.

^44^ Morley et al., op. cit. note 4.

^45^ Gallagher, A., Bousso, R, S., McCarthy, J. et al. (2015). Negotiated reorienting: a grounded theory of nurses' end‐of‐life decision‐making in the intensive care unit. *International Journal of Nursing Studies,* *52*(4), 794–803.

^46^ Morley et al., op. cit. note 5.

^47^ Morley et al., op. cit. note 5.

^48^ Oberle, K. & Hughes, D. (2001). Doctors’ and nurses’ perceptions of ethical problems in end‐of‐life decisions. *J Adv Nurs,* *33*(6), 707–715.

^49^ Dzeng, E. (2017). Navigating the liminal state between life and death: Clinician moral distress and uncertainty regarding new life sustaining technologies. *Americal Journal of Bioethics,* *17*(2), 22–25.

^50^ Ibid: 23.

^51^ April, C. W. & April, M. D. (2016). Understanding moral distress through the lens of social reflective equilibrium. *American Journal of Bioethics,* *16*(12), 25–27.

^52^ Campbell et al., op. cit. note 3.

^53^ April & April, op. cit. note 51, p. 27.

^54^ Ibid.

^55^ Johnstone, M. J. & Hutchinson, A. (2015). 'Moral distress'—time to abandon a flawed nursing construct? *Nursing Ethics,* *22*(1), 5–14.

^56^ April & April, op. cit. note 51.

^57^ Haidt, J. (2003). The moral emotions. In R. J. Davidson, K. R. Scherer, & H. H. Goldsmith, *Handbook of affective sciences*. Oxford University Press. http://faculty.virginia.edu/haidtlab/articles/alternate_versions/haidt.2003.the‐moral‐emotions.pub025‐as‐html.html

^58^ April & April, op. cit. note 51.

^59^ Batho, D. & Pitton, C. (2018). What is moral distress? Experiences and responses. The University of Essex, 1–33.

^60^ Ibid: 7.

^61^ For example, American Association of Critical‐Care Nurses, AACN (2006). Public policy position statement: Moral distress. CA, USA; Canadian Nurses Association (2008). Code of ethics for registered nurses. Ottawa; Hamric, A. (2006). Case study of moral distress. *Journal of Hospice and Palliative Nursing* 2014, *16*(8), 457–463; Thomas, T. & McCullough, L. (2015). A philosophical taxonomy of ethically significant moral distress. *Journal of Medicine and Philosophy,* *40*, 102–120.

^62^ Batho & Pitton, op. cit. note 59, p. 15.

^63^
www.consciencelaws.org

^64^ Hardingham, L. (2004). Integrity and moral residue: nurses as participants in a moral community. *Nursing Philosophy,* *5*, 127–134.

^65^ Batho & Pitton, op. cit. note 59.

^66^ Ibid.

^67^ Ibid.

^68^ Huxtable, R. (2012). *Law, ethics and compromise at the limits of life: To treat or not to treat?* London: Routledge‐Cavendish, pp. 140–141.

^69^ In particular arguments made by Fourie, C. (2015) and Campbell et al. (2016).

^70^ Code, L. (2002). Narratives of responsibility and agency: Reading Margaret Urban Walker’s *Moral understandings*. *Hypatia,* *17*, 156–173.

